# A complete picture of protein unfolding and refolding in surfactants†

**DOI:** 10.1039/c9sc04831f

**Published:** 2019-11-22

**Authors:** Jannik Nedergaard Pedersen, Jeppe Lyngsø, Thomas Zinn, Daniel E. Otzen, Jan Skov Pedersen

**Affiliations:** Interdisciplinary Nanoscience Center (iNANO), Department of Chemistry, Aarhus University Gustav Wieds Vej 14 DK – 8000 Aarhus C Denmark jsp@chem.au.dk; ESRF - The European Synchrotron 38043 Grenoble France; Interdisciplinary Nanoscience Center (iNANO), Department of Molecular Biology and Genetics, Aarhus University Gustav Wieds Vej 14 DK – 8000 Aarhus C Denmark dao@nano.au.dk

## Abstract

Interactions between proteins and surfactants are of relevance in many applications including food, washing powder formulations, and drug formulation. The anionic surfactant sodium dodecyl sulfate (SDS) is known to unfold globular proteins, while the non-ionic surfactant octaethyleneglycol monododecyl ether (C_12_E_8_) can be used to refold proteins from their SDS-denatured state. While unfolding have been studied in detail at the protein level, a complete picture of the interplay between protein and surfactant in these processes is lacking. This gap in our knowledge is addressed in the current work, using the β-sheet-rich globular protein β-lactoglobulin (bLG). We combined stopped-flow time-resolved SAXS, fluorescence, and circular dichroism, respectively, to provide an unprecedented in-depth picture of the different steps involved in both protein unfolding and refolding in the presence of SDS and C_12_E_8_. During unfolding, core–shell bLG-SDS complexes were formed within ∼10 ms. This involved an initial rapid process where protein and SDS formed aggregates, followed by two slower processes, where the complexes first disaggregated into single protein structures situated asymmetrically on the SDS micelles, followed by isotropic redistribution of the protein. Refolding kinetics (>100 s) were slower than unfolding (<30 s), and involved rearrangements within the mixing deadtime (∼5 ms) and transient accumulation of unfolded monomeric protein, differing in structure from the original bLG-SDS structure. Refolding of bLG involved two steps: extraction of most of the SDS from the complexes followed by protein refolding. These results reveal that surfactant-mediated unfolding and refolding of proteins are complex processes with rearrangements occurring on time scales from sub-milliseconds to minutes.

## Abstract 2

The time-resolved study reveals several transition states during SDS-induced unfolding of the protein, as well as under refolding of the protein by the nonionic surfactant C_12_E_8_.

## Graphical abstract

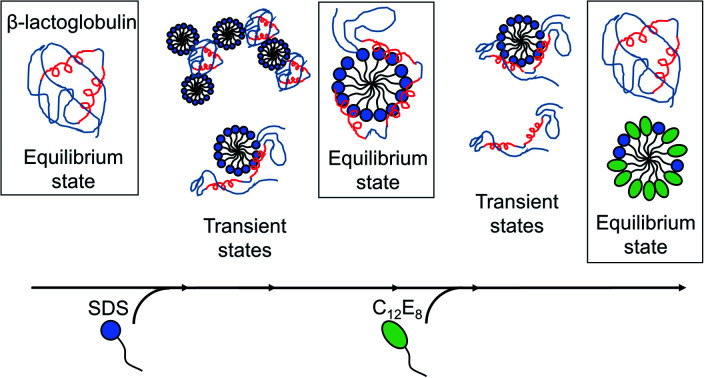

## Introduction

Protein–surfactant interactions have been intensely studied for decades, both because of the richness of different protein conformations induced by surfactants and also because of the importance of protein–surfactant mixtures in many industrial applications such as detergents and cosmetics formulations.^1–3^ Surfactants can either stabilize, destabilize, unfold, or avoid binding to proteins, depending on the protein and surfactant in question. Despite a long history of research, there are many open questions about the structural and mechanistic aspects of the formation of protein–surfactant complexes. Due to their strong and destabilizing interactions with proteins, anionic surfactants (particularly sodium dodecyl sulfate, SDS) have long been a major focus in kinetics^4^ and equilibrium measurements.^5^ Protein–surfactant complexes are not static or uniform; they are sensitive to the absolute concentration of surfactant and protein–surfactant stoichiometries as well as the nature of the surfactant and protein. A large body of work suggests that SDS-denatured proteins consist of nearly intact SDS micelles decorated with partially unfolded protein.^6–10^ While normal globular proteins do not interact to any significant extent with non-ionic surfactants (NIS), membrane proteins require a membrane-like environment and are typically solubilized in their native state by NIS micelles. Further, addition of NIS to SDS–protein complexes leads to the formation of mixed SDS–NIS micelles, which weakens the denaturing potency of SDS.^4^ This mixing facilitates the refolding of many membrane proteins from their SDS-unfolded state^11–14^ and can also be applied to the study of water-soluble proteins. We have recently exploited the formation of SDS–NIS micelles to refold three globular proteins (β-lactoglobulin (bLG), lysozyme, and bovine serum albumin (BSA)) from SDS by addition of the non-ionic surfactant octaethylene glycol monododecyl ether (C_12_E_8_).^10^ C_12_E_8_ extracts SDS from the protein–SDS complexes into mixed micelles, leaving the protein free to refold. Surprisingly, protein unfolding by SDS does not require electrostatic interactions between SDS and the protein, since proteins lacking ionizable side chains are also readily and rapidly unfolded in SDS.^15^ Based on this observation, we have suggested that protein destabilization is driven by access to the hydrophilic–hydrophobic interface on the SDS micelle, due to the small but highly charged sulfate head groups. In contrast, C_12_E_8_ has a large uncharged head group that sterically screens access to the hydrophilic–hydrophobic interface of the micelles, thus giving rise to weak interactions with proteins and a preference for self-assembly.

**Fig. 1:**
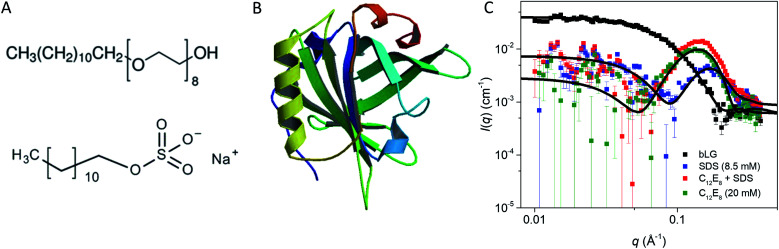
(A) Stick representation of octaethylene glycol monododecyl ether (C_12_E_8_) and sodium dodecyl sulfate (SDS). (B) Crystal structure of bLG (PDB entry 3NPO^16^). (C) Small-angle X-ray scattering data of β-lactoglobulin (bLG), SDS, C_12_E_8_, and C_12_E_8_-SDS mixed micelles as well as model fits to bLG, SDS, and C_12_E_8_ data.

Our previous work on the refolding of SDS-denatured proteins^10^ was carried out under equilibrium conditions. However, protein unfolding and refolding kinetics provide further insight into the different steps occurring during unfolding and refolding. Unfolding is carried out by simple mixing of the native protein with SDS.^2,4,17^ Refolding from the SDS-denatured state can for example be accomplished by rapid removal of SDS with alpha-cyclodextrins^18^ or by dilution with non-ionic surfactants. The latter approach is used in the present study, where bLG was unfolded by addition of SDS and subsequently refolded by addition of C_12_E_8_ (Fig. 1A). bLG's secondary structure consists mainly of β sheets (Fig. 1B) but the protein converts to a predominantly α-helical state upon SDS binding,^10^ leading to large changes in far-UV CD spectra. This makes bLG particularly suited for a detailed study as it allows the combination of complementary spectroscopic techniques and SAXS. Hitherto unfolding and refolding have only been followed at the protein level and we still lack an understanding of how surfactant and protein cooperate to affect unfolding and refolding. Here we address this deficiency by carrying out both unfolding and refolding at the millisecond timescale using stopped-flow mixing in combination with three different experimental techniques. Changes in bLG secondary and tertiary structure were monitored using circular dichroism (CD) and Trp fluorescence, while changes in the overall architecture of the protein–surfactant complexes were followed by synchrotron small-angle X-ray scattering (SAXS). Thanks to the intense beamlines at synchrotrons, it has been possible to obtain time-resolved SAXS data (down to sub-millisecond) for several decades.^19^ Stopped-flow SAXS has been used to monitor *e.g.* formation of inorganic nanoparticles,^20,21^ conformational changes in RNA^22^ and in proteins,^23,24^ formation and rearrangements of surfactant micelles,^25,26^ and early stages of protein aggregation.^27^ However, to the best of our knowledge, the formation and rearrangements of protein–surfactant complexes have not been subjected to this type of scrutiny. SAXS is highly appropriate in this regard, as it provides information on the overall shape of the protein–surfactant complex rather than reporting solely on the protein structure, and thus provides a more complete picture of the structural changes both at the protein and surfactant level associated with unfolding and folding.

Due to its role as a small-molecule transporter, folded bLG contains several hydrophobic binding sites. Monomeric SDS can bind to these sites, stabilizing the native state,^28^ though higher concentrations of SDS denature bLG in a cooperative fashion. Using known SDS–bLG binding stoichiometries^29^ as well as sufficiently high C_12_E_8_ : SDS ratios to refold bLG,^10^ we report different intermediate states, which transiently occur during both un- and re-folding of bLG. Using SAXS, low resolution structures of these intermediates have been determined, revealing how surfactants bind to bLG in several steps. Our work provides deeper insight into the structural changes occurring at the protein–surfactant level during conformational changes induced by surfactants.

## Results

### Structural characterization of initial components

We start by using SAXS to analyze the structures of the three individual components, *i.e.*, SDS micelles, C_12_E_8_ micelles, and natively folded bLG (Fig. 1C). Depending on temperature, pH, and protein concentration, bLG can exist either as monomer, dimer, or octamer.^30^ Under our experimental conditions (2 mg mL^−1^, pH 7.0), bLG is expected to exist as 75% dimer and 25% monomer.^31,32^ To ascertain this more directly, we used the monomeric crystal structure of bLG (PDB entry 3NPO^16^) and the dimeric crystal structure of bLG (PDB entry 1BEB^33^) to create, respectively, the scattering of monomers and dimers. A linear combination of these two calculated scattering curves was fitted to the SAXS data. The best fit was for a contribution of 77% dimer and 23% monomer (Fig. 1C) in excellent agreement with previous measurements.^31,32^ The scattering data from both SDS and C_12_E_8_ micelles could be fitted on absolute scale by a model of core–shell ellipsoids of revolution (spheroids). For SDS and C_12_E_8_ micelles, respectively, this led to a core long-axis radius of 22.7 ± 0.4 Å and 21.0 ± 1.0 Å, shell thicknesses of 5.5 Å and 15.0 Å, core axis ratios of 0.60 ± 0.03 and 0.64 ± 0.03 (*i.e.*, short axes for the micellar cores of 13.6 ± 0.4 Å and 13.4 ± 0.6 Å) and aggregation numbers of 85 and 70. The similarity in core sizes for SDS and C_12_E_8_ reflects the 12-carbon alkyl chain length shared by the two surfactants, while the large difference in shell thicknesses arises from SDS's very small sulfate head group, contrasting with C_12_E_8_'s larger eight ethylene glycol (–O–CH_2_CH_2_–) groups.

### bLG rearrangements occur in several steps

**Fig. 2:**
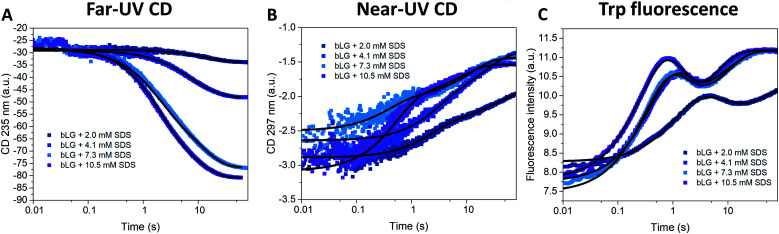
Unfolding kinetics of bLG followed by mixing bLG with different concentrations of SDS and tracked with (A) far-UV CD at 235 nm, (B) near-UV CD at 297 nm, and (C) fluorescence at 355 nm. Fits with exponential decay functions are plotted and the corresponding half times (*t*_1/2_) from fitting are shown in Table 1.

Changes in bLG's structure at different SDS concentrations have previously been determined by CD.^10^ Addition of SDS shifts the far-UV CD spectrum from a clear minimum at 217 nm (characteristic of β sheets) to a double minimum at 208 and 222 nm, consistent with a change to a mainly α-helical structure (Fig. S1†). Near-UV CD highlights the loss of tertiary structure; a peak at 294 nm in the folded state completely disappears upon unfolding^10^ (Fig. S1†). Changes in tertiary structure can also be followed with tryptophan (Trp) fluorescence, which shows a marked increase in intensity (but little change in peak position) as SDS is added (Fig. S1†). Based on these indications, we turned to stopped-flow investigations (deadtime 5 ms) to follow the kinetics of the structural changes of bLG upon SDS unfolding using both near/far-UV CD and Trp fluorescence (Fig. 2). Unfolding was investigated at four different SDS concentrations, corresponding to characteristic stages of denaturation as determined by isothermal titration calorimetry (ITC).^29^ The highest concentration (10.5 mM SDS) corresponds to the final titration stage where the protein is saturated with SDS but no free micelles has started to form, while the three lower SDS concentrations (2.0, 4.1, and 7.3 mM SDS) were below saturation. Fig. 2 (recorded over 60–80 s) shows that both secondary and tertiary structural changes reached completion within ∼1 min. For bLG unfolded with 10.5 mM SDS, a large change in the far-UV CD signal was observed, arising from the conversion of β sheet into α-helix secondary structure. The shift diminished as less SDS was added, consistent with a gradual change in the secondary structure as more and more SDS is added (Fig. 2A and S1†). Near-UV CD and Trp fluorescence, which both report on tertiary structure, showed similar changes for the three highest SDS concentrations (4.1, 7.3, and 10.5 mM); only the lowest SDS concentration of 2.0 mM failed to abolish tertiary structure completely (Fig. 2B, C, and S1†). Both far- and near-UV CD data could be fitted with double exponential decays. In contrast, Trp kinetics required three decays: an overshoot within the first 10 s, followed by a small decrease and finally an increase again to a plateau (Fig. 2C). Attempts to use fewer decay functions significantly reduced the quality of the fits. This suggests the transient accumulation of structural intermediates rather than a simple two-state transition. The half-lives (*t*_1/2_) from these decays are listed in Table 1 and they showed a reasonable correspondence between the three different techniques. Note that in general far-UV CD only detected the two slowest phases measured by Trp fluorescence, whereas near-UV CD detected both the fastest and the slowest Trp phase.

**Table 1:** Kinetic parameters from stopped-flow CD, Trp fluorescence, and SAXS

	Unfolding												Refolding					
	bLG + 10.5 mM SDS			bLG + 7.3 mM SDS			bLG + 4.1 mM SDS			bLG + 2.0 mM SDS			*χ*_SDS_ = 0.45			*χ*_SDS_ = 0.30		
	*t*_1/2,1_ (s)	*t*_1/2,2_ (s)	*t*_1/2,3_ (s)	*t*_1/2,1_ (s)	*t*_1/2,2_ (s)	*t*_1/2,3_ (s)	*t*_1/2,1_ (s)	*t*_1/2,2_ (s)	*t*_1/2,3_ (s)	*t*_1/2,1_ (s)	*t*_1/2,2_ (s)	*t*_1/2,3_ (s)	*t*_1/2,1_ (s)	*t*_1/2,2_ (s)	*t*_1/2,3_ (s)	*t*_1/2,1_ (s)	*t*_1/2,2_ (s)	*t*_1/2,3_ (s)
Far-UV CD		1.19 ± 0.02	5.98 ± 0.17		1.29 ± 0.11	7.50 ± 0.27		3.08 ± 0.12	10.9 ± 0.2			10.5 ± 0.3	0.50 ± 0.01	17.2 ± 0.1		0.18 ± 0.01	3.9 ± 0.2	
Near-UV CD	0.36 ± 0.01		5.39 ± 0.04	0.27 ± 0.01		8.14 ± 0.06	1.86 ± 0.02		15.8 ± 0.1	1.52 ± 0.05		24.4 ± 2.1		14.9 ± 0.1	377 ± 2		5.9 ± 0.3	72 ± 1
Trp fluorescence	0.23 ± 0.01	0.75 ± 0.02	3.84 ± 0.21	0.25 ± 0.01	1.00 ± 0.04	6.22 ± 0.51	0.36 ± 0.02	0.69 ± 0.04	6.78 ± 0.41	0.85 ± 0.06	6.58 ± 0.43	26.6 ± 3.2		19.5 ± 0.2	448 ± 3		6.2 ± 0.3	71 ± 1
SAXS	0.36 ± 0.03^a^	1.38 ± 0.19^b^		0.51 ± 0.04^a^	1.17 ± 0.15^b^		0.97 ± 0.10^a^	1.25 ± 0.20^b^		0.84 ± 0.08^a^					357 ± 32^c^		1.3 ± 0.2^c^	92 ± 8^c^
															242 ± 19^d^		1.8 ± 0.32^d^	101 ± 23^d^
															1110 ± 94^e^			385 ± 21^e^

a Based on number of proteins per complex.

b Based on core displacement.

c Based on fraction of native protein.

d Based on fraction of random coil.

e Based on fraction of complex.

### SDS initially binds mainly on one side of bLG and bLG slowly wraps around the micelle

**Fig. 3:**
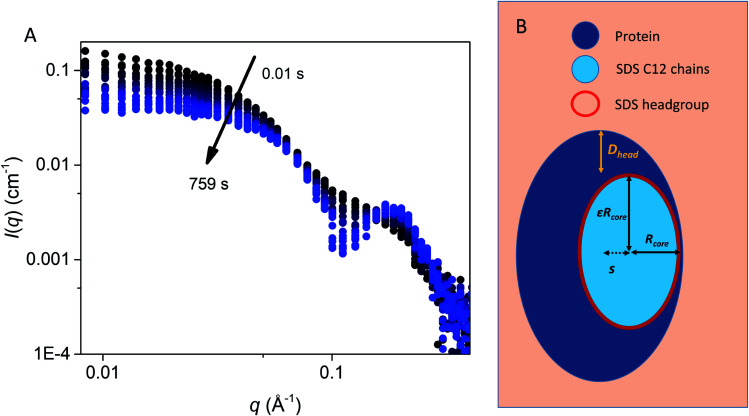
(A) Stopped-flow SAXS data of bLG mixed with 10.5 mM SDS at a time resolution of ∼4 ms and measured for 12.5 min. The arrows show the progression over time. (B) The model used for analysis of the SAXS data for unfolding of bLG mixed with SDS with *R*_core_ representing the core radius, *ε* representing the axis ratio, *D*_head_ the head group thickness, and an adjustable core offset represented by *s*. The model is based on Pilz *et al.*^34^

To elucidate changes in the overall shape of the protein–detergent complexes, we recorded stopped-flow time-resolved SAXS with a deadtime of ∼4 ms (Fig. 3 and S2†). We note that shearing during the mixing process and transfer to the measuring cell do not directly influence the aggregation state of the surfactant micelles.^25,26^ We have previously shown that the final SDS–bLG complexes are core–shell structures in which a central SDS micelle is surrounded by a shell of partially unfolded protein.^10^ The signal right after mixing for the first measurement is much higher than that for the final state, in particular for low *q* (where *q* is the modulus of the scattering vector). Further, data at early time points have a much smoother and monotonic dependence on *q*. The high intensity at low *q* shows that the early protein–bLG complexes are much larger than in the final state. This is also reflected in the corresponding distance distribution functions, *p*(*r*) (Fig. S3†), for which the maximum diameter *D*_max_ is large initially and decreases over time. Furthermore, the radius of gyration, *R*_g_, and the forward scattering, *I*(0), determined from Guinier fits (Fig. S4†) both show a decay as a function of time. A smooth dependence of the intensity on *q* is typical of the co-existence of multiple different states.^10^ We initially attempted to describe the data using simply linear combinations of the following states: (1) the final unfolded protein complex, (2) free micelles, and (3) free (folded) protein (Fig. S5†). If such a linear combination can describe the data, it suggests that the denaturing and subsequent unfolding is cooperative, so that the protein is either in one state or the other. However, it was not possible to describe the data with this simple model at the initial ∼0.5 s as seen by a high *χ*^2^ value at these early time points (Fig. S5†). Consistent with the spectroscopic data in Fig. 2, this indicates that unfolding is not a simple two-state process but rather involves intermediate structures, whose SAXS profiles differ both from the final complex and the initial species (Fig. S5†). Intuitively, a simple multi-step binding of SDS to bLG might involve initial attachment of SDS to one side of the protein (perhaps guided by long-range electrostatic interactions), followed by further protein unfolding, which allows bLG to wrap more symmetrically around the SDS micelle. To model this, we used a modified ellipsoidal core–shell model^6^ for the SDS–protein complex in which the centers of the ellipsoidal core and the outer surface of the complex do not coincide. The model, which is based on the expressions given by Pilz *et al.*,^34^ contains an adjustable core offset, *s*, perpendicular to the long axis (Fig. 3). This enables the description of an anisotropic distribution of the protein on the micellar surface. To describe the initial aggregation that occurs just after mixing, a random flight structure factor was multiplied on the scattering form factor of the complex.^35,36^

**Fig. 4:**
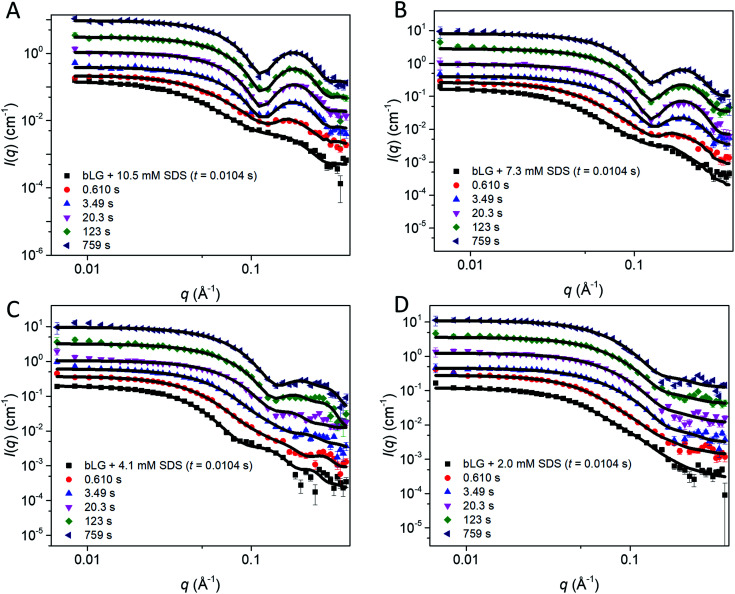
Stopped-flow SAXS data of bLG mixed with (A) 10.5 mM SDS, (B) 7.3 mM SDS, (C) 4.1 mM SDS, and (D) 2.0 mM SDS at selected times through the series. Lines represent fits to the core–shell model with an adjustable core offset. For clarity, the data has been scaled with a factor of 3 for every displayed time step.

**Fig. 5:**
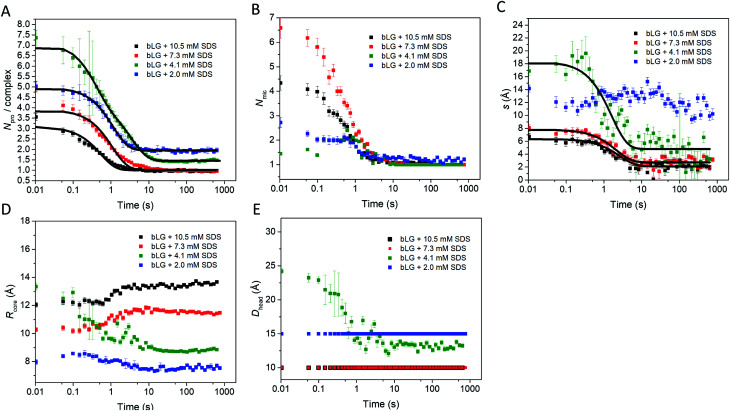
Structural model parameters obtained from fitting the core–shell model with an adjustable core offset to the stopped-flow unfolding SAXS data. The obtained parameters are: (A) number of proteins per complex, (B) number of micelles per complex, (C) core displacement, (D) radius of the core, and (E) size of the head group. For (A and C), single exponential decay functions were fitted to the data and the half times (*t*_1/2_) are displayed in Table 1. The core displacement at 2.0 mM SDS could not be fitted with an exponential function.

We first tested this on the end state of the protein (*i.e.*, SAXS profiles recorded after *ca.* 759 s) with four different SDS concentrations (Fig. 4, results summarized in Table 2). Modelling was done on absolute scale, *i.e.*, the total concentrations of SDS and protein were kept constant at predetermined values. With increasing amounts of SDS, we saw a gradual decrease in head group thickness (*D*_head_), core axis ratio (*ε*), proteins per complex (*N*_pro_), and core displacement (*s*), as well as an increase in the core radius (*R*_core_). For 4.1 and 2.0 mM bound SDS, the best fits at the end of the unfolding process are obtained for small asymmetrical structures with 1–2 bLG molecules per complex (Fig. 5A). In contrast, at 10.5 and 7.3 mM SDS, the SAXS data are fitted well with a model where SDS forms a micelle in the center of the complex surrounded by a single protein molecule. Clustering of micelles (*N*_mic_) to form higher-order complexes are sometimes seen,^5^ but was only present at 2.0 mM SDS in the end states. Our core–shell model, which allows an asymmetric distribution, reproduces the data well at all SDS concentrations. However, at low SDS concentrations, other models may be considered relevant since the SDS contribution is generally low and may be insufficient to form one micelle per protein with the result of small clusters forming containing more proteins per SDS cluster.

**Table 2:** Fitting parameters from fitting a core–shell model with a displaced core to SDS-unfolded bLG end state SAXS data

	*D*_head_ (Å)	*R*_core_ (Å)	*ε*	*N*_mic_	*D*_mic_ (Å)	*C*_SDS,total_ (mM)	*C*_SDS,free_ (mM)	*N*_pro_	*s* (Å)
2.0 mM SDS	15.0 ± 0.3	7.6 ± 0.1	3.4 ± 0.5	1.15 ± 0.04	50^b^	2.0^c^	0.7^c^	1.96 ± 0.11	7.4 ± 2.8
4.1 mM SDS	13.2 ± 0.3	8.8 ± 0.1	3.4 ± 0.3	1.0^a^	50^b^	4.1^c^	1.8^c^	1.48 ± 0.02	3.2 ± 1.5
7.3 mM SDS	10.2 ± 0.1	11.5 ± 0.1	2.1 ± 0.1	1.0^a^	50^b^	7.3^c^	2.6^c^	0.97 ± 0.01	2.1 ± 0.4
10.5 mM SDS	10.1 ± 0.1	13.7 ± 0.1	1.8 ± 0.1	1.0^a^	50^b^	10.5^c^	3.7^c^	0.98 ± 0.01	1.9 ± 0.3

a The parameter converged at 1.0 and was locked.

b The parameter was locked at a reasonable value determined from the size of the complexes.

c Locked at known or estimated concentrations from Hansted *et al.*^29^

In the next step, all data from the full time series of all four SDS concentrations were individually fitted by the model (Fig. 4, 5, and S6†). Good fits to the SAXS data were obtained throughout the series and therefore, as a simple quantitative representation of the structural changes that occur over time, we consider this model sufficient. For simplicity, several parameters which did not change markedly over time towards the end of the series or converged at some value were locked at average (meaningful) values. Specifically, the distance between the protein-decorated micelles in the random flight clusters (*D*_mic_) was kept constant at 50 Å at all SDS concentrations and *ε* was kept constant at the values obtained for the equilibrium end state (1.8–3.4 depending on SDS concentration). The head group thickness *D*_head_ and the micellar core radius *R*_core_ did not fluctuate markedly. Satisfactory fits were obtained when *D*_head_ was kept constant, based on equilibrium data, either at 10 Å (2.0 mM SDS) or 15 Å (7.3 and 10.5 mM SDS). The only exception was at 4.1 mM SDS where best fits were obtained when *D*_head_ decreased from an initial value of 24.2 ± 0.4 Å to an end value of 13.2 ± 0.3 Å. Attempts to keep *D*_head_ constant for the sample containing 4.1 mM SDS resulted in poor fitting. Similarly, *R*_core_ underwent a significant reduction from 13.3 ± 0.2 Å to 8.8 ± 0.1 Å in the same time period. These restrictions led to the values for the off-centered core–shell model displayed in Fig. 5. Several trends can be observed: at all SDS values there is a high amount of initial clustering (proteins/micelles and micelles per cluster) which decreases over time (Fig. 5A and B). Above 2.0 mM SDS, there is a large initial core displacement which likewise decays over time (Fig. 5C). At 2.0 mM SDS, this value stays at a constant high value (∼12 Å), probably due to limited unfolding of the protein (*cf.*Fig. 2), which prevents the protein from attaching properly all the way around the SDS cluster. It might be expected that free micelles or free protein could contribute to initial signals. However, including contributions from these species did not improve fitting and we therefore assumed that all SDS associated with protein within the first 4 ms. This is not unreasonable; under stopped-flow conditions, 1 mg mL^−1^ bLG (∼27 μM) is present, which at a hypothetical (modest) second-order protein–SDS micelle association constant of 10^7^ M^−1^ s^−1^ would correspond to an apparent rate constant of binding of 270 s^−1^ (or a half-life of 2.5 ms).

To allow direct comparison with the changes in secondary and tertiary structure, we fitted single exponential functions to the resulting SAXS fitting parameters (Table 1 and Fig. 5). The number of proteins per complex and the core displacement were used for these fits since these parameters changed markedly with time. The samples with 10.5 and 7.3 mM SDS form typical core–shell structures and had similar behavior in their kinetic parameters. SAXS *t*_1/2_-values (0.36–0.51 s) corresponded well to the fast phases observed by near-UV CD (*t*_1/2_ = 0.27–0.36 s) and Trp fluorescence (*t*_1/2_ = 0.23–0.25 s). Near-UV CD and Trp fluorescence are both sensitive to environmental changes around the aromatic amino acids as well as changes in aggregation state. Initial binding of SDS to protein could very well cause changes in this environment. The initial change was followed by secondary structural changes as seen by far-UV CD (*t*_1/2_ = 1.19–1.29 s) in the range where the SAXS model gives changes in the core displacement (*t*_1/2_ = 1.17–1.38 s). The core displacement represents the continued unfolding process where the protein wraps around the SDS micelle; changes in secondary structure are likely necessary to form a stable complex with the protein uniformly surrounding the micelle. In this range, an additional decay phase is also seen for Trp fluorescence (*t*_1/2_ = 0.75–1.00 s), indicating that the Trp environment is still changing. The slow phase (*t*_1/2_ = 4–27 s) seen by far-UV CD, near-UV CD, and Trp fluorescence is not reflected in the SAXS results. We attribute this to structural protein rearrangements on the micelle surface that do not affect overall complex shape and symmetry.

### Intermediate species are present upon refolding by addition of C_12_E_8_ to SDS and the complete refolding process is slow

**Fig. 6:**
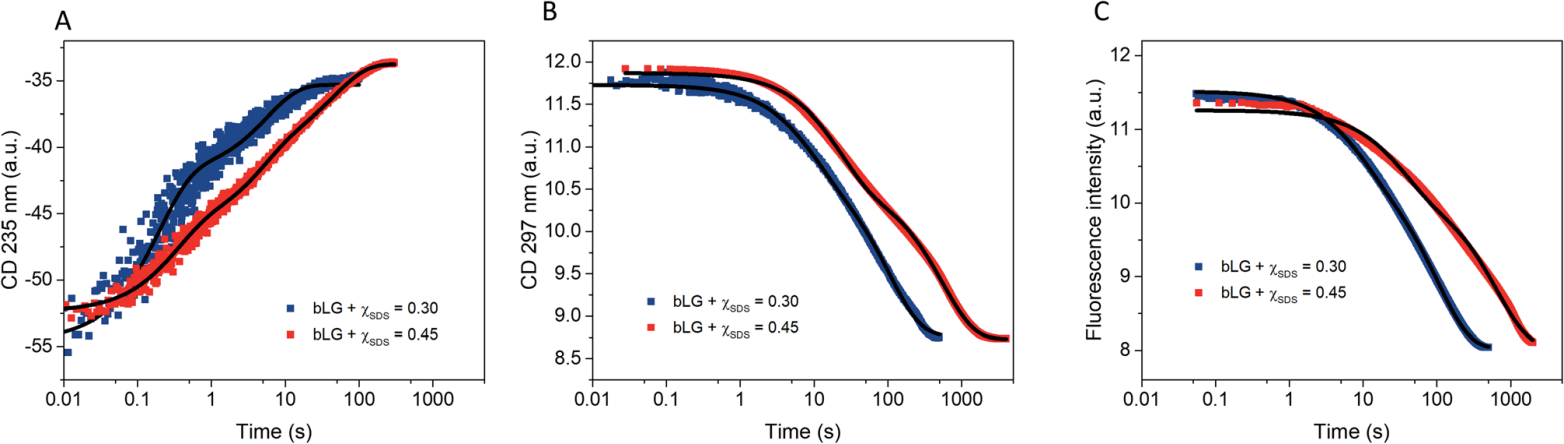
Refolding kinetics followed by mixing bLG-SDS complexes with two different mol ratios of C_12_E_8_ tracked with: (A) far-UV CD at 235 nm, (B) near-UV CD at 297 nm, and (C) fluorescence at 355 nm. Fits with exponential decay functions are plotted and the corresponding half times (*t*_1/2_) from fitting are shown in Table 1.

Having completed our analysis of the unfolding steps, we now turn to refolding. In this process, SDS-denatured bLG is mixed with the non-ionic surfactant C_12_E_8_ and the process is again first monitored with far- and near-UV CD as well as Trp fluorescence (Fig. 6). Concentrations of C_12_E_8_ were chosen based on equilibrium data^10^ where refolding was achieved at a SDS mole fraction (*χ*_SDS_) of 0.45 as seen by near/far-UV CD and SAXS. We used C_12_E_8_ concentrations only just sufficient to allow bLG to refold (*χ*_SDS_ = 0.45) as well as concentrations well in excess of this threshold (*χ*_SDS_ = 0.30). Kinetics from all three techniques could be fitted individually with double exponential functions. In combination, they showed changes occurring on three overlapping timescales. The kinetics at *χ*_SDS_ = 0.30 were 3–6-fold faster than at *χ*_SDS_ = 0.45, but otherwise showed similar behavior (Table 1). The fastest process (*t*_1/2_ = 0.18–0.5 s) was only seen by far-UV CD, an intermediate process (*t*_1/2_ = 5–15 s) was detected with all three techniques, while the slowest process (presumably reacquisition of the native tertiary structure) was much slower (*t*_1/2_ = 71–448 s).

As with unfolding, we complemented these data with SAXS analyses. To analyze the SAXS data acquired during refolding, we tried several different approaches. Making the minimalist assumption that unfolding by SDS is a single-track process rather than occurring in several parallel processes, the simplest approach is to model the refolding population as consisting of: (1) protein–SDS complexes that gradually change into (2) the refolded monomer structure by reducing the amount of SDS bound to bLG and (3) mixed SDS-C_12_E_8_ micelles. However, this approach only gave satisfactory fits when unreasonable fitting parameters were used (data not shown). Instead, we attempted a linear combination model of well-defined species. In the first attempt, we combined measured data sets of: (1) the unfolded bLG-SDS complex, (2) folded bLG (77% dimer/23% monomer), (3) SDS micelles, and (4) mixed SDS-C_12_E_8_ micelles. We noted that the scattering curves from SDS and mixed micelles are very similar (Fig. 1C), thus resulting in large correlations between the scale factors of the two contributions in the fits. Therefore, only mixed micelles were used in the rest of the analysis. The linear combination described the data very well with *χ*^2^ values around 2.0 for most curves (Fig. S7A†). However, when summing the scale factors that represents the concentrations of the two protein contributions, it was apparent (Fig. S7B†) that the protein total mass in the samples was not constant. This indicates that an additional protein structure with a different scattering curve needs to be included. It could be observed from the intensity levels and the fits to the first SAXS data sets in each series that this protein structure had to be monomeric, since the scale factor was below one (Fig. S7B†) and structures larger than the monomer would only decrease the summed scale factor. We note that some changes in the bLG monomer–dimer equilibrium can be expected during the refolding process, so therefore we attempted using a scattering curve calculated from the monomeric bLG PDB structure. However, this did not give satisfactory fits. As a next attempt we assumed that the structure was fully unfolded and therefore described it by the scattering of a Gaussian chain. For the refolded species, we returned to using the experimental SAXS curve, although, as mentioned, some change in the monomer–dimer equilibrium could occur. As shown in the following, the combination with the Gaussian chain scattering for the unfolded protein and conservation of the total protein mass gave satisfactory fits. We note that the system is expected to end up with the same monomer–dimer equilibrium as the pure sample as demonstrated in the previous study of the equilibrium structures^10^ and considering that good fits could be obtained without the addition of a monomeric folded species, it was reasonable to omit this in the final fits.

**Fig. 7:**
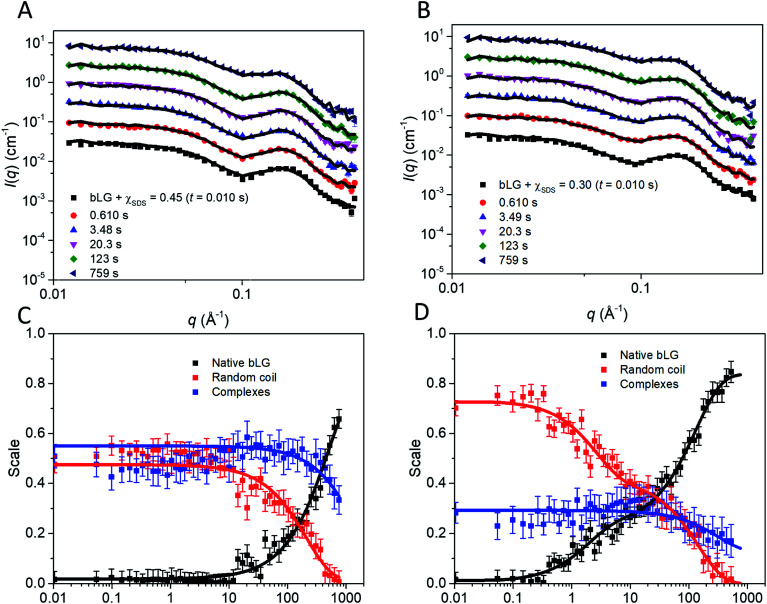
Stopped-flow SAXS data of bLG-SDS complexes mixed with C_12_E_8_ to (A) *χ*_SDS_ = 0.45 and (B) *χ*_SDS_ = 0.30. For clarity, the data has been scaled with a factor of 3 for every displayed time step. Contributions from the scales of the various protein species for data with (C) *χ*_SDS_ = 0.45 and (D) *χ*_SDS_ = 0.30. In (C and D) exponential fits are plotted and the corresponding half times (*t*_1/2_) from fitting are shown in Table 1.

In the fit of the two refolding series, conservation of protein using constraints on scale factors of the various contributions was imposed. The size of the random-coil chains in terms of the radius of gyration could in general not be optimized during the fit as it was not sufficiently stable. Therefore it was kept at 30 Å, except for the first few frames, where it had to be slightly larger (Fig. S8†). The fits to the SAXS data with this approach were satisfactory (Fig. 7A, B and S9†). The development of the scales (mass fractions) (Fig. 7C and D) of the various protein species showed structural changes occurring in more than one step (Table 1). Already by the time the first frame is recorded (∼10 ms), a large fraction of the complexes has converted to the unfolded random-coil state, and this fraction increases as more non-ionic surfactant is added (*χ*_SDS_ = 0.3). There is hardly any development in the species distribution within the first second, but after this time, there is a gradual conversion of the random-coil structure into natively folded protein, while the amount of complexes stays constant. At *t*_1/2_ ≈ 385 s for *χ*_SDS_ = 0.3 and *t*_1/2_ ≈ 1110 s for *χ*_SDS_ = 0.45, the amount of complexes decreases as they are converted into natively folded protein. The refolding process was only followed for 500–800 s with SAXS, while the data suggest that longer measurement times could have been relevant to obtain equilibrium structures. However, longer measurement times were not feasible due to limited synchrotron beam access. CD and fluorescence kinetics generally follow the SAXS timescales (Table 1). Secondary structure changes occur mainly in the short timescales (<4–17 s), while tertiary structural changes generally occur during the last slow step where native protein accumulates, in good agreement with the suggested SAXS model.

## Discussion

### Asymmetrical core–shell complexes are rapidly formed upon unfolding in SDS

**Fig. 8:**
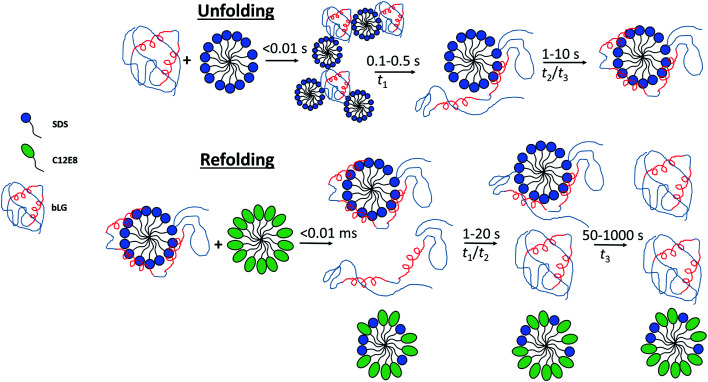
Sketch showing the suggested unfolding and refolding scheme when bLG is unfolded with SDS and subsequently refolded with C_12_E_8_. Unfolding occurs in three steps with an initial clustering of SDS and protein within the deadtime of the experiment, followed by disaggregation of the complexes into individual SDS micelles with asymmetrically distributed protein, as seen by changes in Trp fluorescence, near-UV CD, and SAXS. In the last step, the protein is redistributed more symmetrically around the SDS micelle, as seen with changes in SAXS, near/far-UV CD, and Trp fluorescence. Refolding is somewhat more complex and slow and also involves three steps. Within the deadtime, a fraction of the protein dissociates from the surfactant micelles while the rest remains in complex, as observed by SAXS. Next, the unfolded protein is folded to a native state and finally the protein still in complex dissociates and refolds. α-Helices are shown in red and two α-helices represent a structure with native-like secondary structure, while three helices represent structures with increased α-helical content. Note that for the refolding process there are additional changes in aggregation state of some of the species, which for simplicity are not indicated in the figure.

By combining SAXS, CD, and fluorescence, we provide a detailed picture of how a protein rearranges during the unfolding and refolding process induced by SDS and C_12_E_8_ surfactants. Unfolding kinetics of bLG with SDS revealed that the unfolding process consists of several steps. For saturated SDS–bLG binding, we propose an unfolding scheme as shown in Fig. 8 consisting of three steps. First, SDS binds to bLG rapidly within the deadtime of the SAXS measurement (∼4 ms), resulting in clustering of bLG and SDS into large complexes. Within the next 100–500 ms, the formed SDS micelles disaggregate into single micelles with bound protein that starts to change its tertiary structure while the secondary structure is still intact. By ∼1 s, bLG begins wrapping more uniformly around the micelle, resulting in an increased α-helical structure of bLG. On the timescale of 1–10 s, both secondary and tertiary structural changes can be observed by CD and fluorescence without notable changes in the SAXS data. At this point, bLG is approximately uniformly distributed around the micelle while minor rearrangements of the protein occur to optimize micelle binding.

### Protein redistribution around the SDS micelle promotes formation of α-helices

Molecular dynamics simulations of the globular protein α-lactalbumin in presence of negatively charged oleate micelles show how the protein is bound on one side of the micelle and gradually unfolds to finally distribute more uniformly around the micelle.^37^ In the α-lactalbumin simulations, binding and redistribution of the protein around the micelle occurred in a few μs, which is much faster than the timescale for bLG redistribution on the micelle which takes seconds. This likely happens in the simulation due to imposed coarse-graining as well as the forced destabilization of the protein, assuring that unfolding is achieved on a short timescale. The final α-lactalbumin–oleate complexes did not have the α-lactalbumin uniformly distributed on the micelle but could still accurately reproduce the corresponding SAXS data. In the case of bLG-SDS complexes, a somewhat uneven distribution of protein surrounding the micelle is also likely, which is evident from model fitting to the bLG-SDS SAXS data where the core at all SDS concentrations was displaced in the steady state measurements (Table 1). A change in secondary structure from mainly β sheet to predominantly α helix upon thermal unfolding of bLG has been reported,^38–40^ suggesting that loss of tertiary structure stabilizes a more α-helical state of bLG. To elucidate a possible general trend, we are currently investigating other proteins with mainly β-sheet structure to see if their structure is likewise altered to a more α-helical-rich structure upon unfolding by SDS.^41^

### Removal of SDS happens through several steps during refolding

Our combined data from fluorescence, CD, and SAXS allow us to propose a scheme for the refolding of bLG with C_12_E_8_ from its SDS denatured state (Fig. 8). The relatively slow extraction of SDS by C_12_E_8_ provides a convenient time window to follow the various processes associated with regrouping and refolding of the protein–surfactant complex. Refolding is overall much slower than unfolding, and both unstructured monomeric bLG and SDS–bLG complexes are observed from the beginning. Best fits were obtained using a contribution from an unfolded species. This suggests that a large fraction of the complexes are disrupted during the turbulent mixing process, so that they are converted into an unfolded state (which contains little SDS). The degree of disruption of the complexes is largest for the sample with highest amount of added non-ionic surfactant, which suggests that the non-ionic surfactants plays a key role in this disruption. To obtain good fits to the data, the radius of gyration of the (partly) unfolded protein contribution had to be decreased during the first 0.1–0.3 s. The changes in secondary structure seen at this time possibly arise from incomplete refolding of the partly unfolded protein and explains why *R*_g_ for the random coil contribution rapidly decreases initially (Fig. S8†). Further conversion into natively folded protein is only significant after 1–10 s. It cannot be ruled out that this refolding is more gradual with occurrence of additional secondary structure, which eventually folds into the native state; however, the chosen basis functions are sufficient for obtaining good fits. The slowest process, which occurs after 30–300 s is identified as the conversion of proteins in the remaining complexes into the native state. The best evidence for this is that this process corresponds to the complete regain of the near-UV CD signal, which is highly sensitive to the correct native fold, since it requires immobilization of aromatic residues through well-defined interactions with other side chains which are only found in the final native state.

### Unfolding and refolding in surfactants are slow processes

Using stopped-flow SAXS together with complementary information on secondary and tertiary structural changes, it has been possible to obtain a deeper understanding of the rearrangements occurring during both the unfolding and refolding processes induced by surfactants. This approach has previously been used to monitor the unfolding and refolding of bLG at pH 3.2 using urea for unfolding and subsequent dilution of urea for refolding.^42^ Here, the integrated SAXS intensities were followed and it was shown that unfolding could be fitted with a single exponential (*t*_1/2_ = 0.96 s) while a double exponential (*t*_1/2_ = 0.7 s and 6.9 s) was needed for refolding. Furthermore, a burst phase was identified (<21 ms) where most structural changes that occurred was within the deadtime of the instrument. The use of integrated SAXS intensities, rather than a rapid succession of individual SAXS data, limits the structural information obtained with this approach. However, advances in X-ray flux allowed us to record reasonable data in the full *q* range with a concomitant gain of insight into structural changes occurring during both unfolding and refolding. Slow refolding from the SDS-denatured state compared to that of the urea-unfolded state is likely because of a stronger binding affinity of SDS to bLG compared to that of urea. bLG has SDS binding pockets and removal of the last few SDS molecules is expected to be particularly slow. In the analysis of the SAXS data, binding of single SDS molecules to bLG is not visible because of the low size and contrast of SDS; unfolded bLG might thus take longer to fold because of residual binding of low amounts of SDS. Even though refolding of bLG by extraction of SDS is very different than *e.g.* pH jump^43^ or urea jump^42^ experiments, it is noteworthy that all these refolding processes show double exponential kinetics.

### Unfolding and refolding with surfactants is not accessible with all-atom simulations

Molecular dynamics studies focusing on unfolding of protein by SDS often conclude that SDS alone is insufficient to unfold a protein.^44,45^ However, the full unfolding process, as observed in the current study, takes seconds, which is not accessible with all-atom simulations at the moment of writing. In order to follow the unfolding with molecular dynamics, destabilization of the protein through *e.g.* heat, modified force fields, or very high concentrations of surfactant are therefore necessary to speed up the process.^37,44,46^ The refolding process is even slower than the unfolding process and would require other designs to follow with molecular dynamics.

## Conclusions

By combining SAXS, CD, and Trp fluorescence, we have obtained structural insights into the kinetics of unfolding and refolding of bLG using surfactants. The unfolding by SDS is a homogeneous process, in the sense that it can be described by a single species in the SAXS modelling, in which the protein is initially asymmetrically distributed around the micelle, and then becomes increasingly symmetrically distributed as a function of time. The refolding by addition of non-ionic surfactant is a more heterogeneous process during which several protein species are present. The initial mixing disrupts a fraction of the complexes and partially unfolded protein coexists with SDS–protein complexes. The partly unfolded protein then converts into a natively folded state, followed by refolding of the remaining protein from the complexes.

## Materials and methods

### Materials

Sodium dodecyl sulfate (SDS, ≥99%), octaethylene glycol monododecyl (C_12_E_8_, ≥98%), β-lactoglobulin (bLG, >90%), and all buffer components were from Sigma Aldrich (St. Louis, MO). All experiments were carried out in duplicates or more.

### Stopped-flow kinetics

A Chirascan spectrophotometer with stopped-flow accessory (Applied Photophysics, Leatherhead, Surrey, UK) equipped with a xenon lamp was used to follow structural changes over time. Circular dichroism (CD) and Trp fluorescence were used as detection methods in a 10 mm path length setup at 24 °C. Near-UV CD was followed at 297 nm with a 2 nm bandwidth, far-UV CD at 235 nm with a 1 nm bandwidth, and Trp fluorescence through excitation at 283 nm with a 2 nm bandwidth and emission measured using a 355 nm cut-off filter. All solutions were mixed at a 1 : 1 volume ratio and final protein concentrations were 2 mg mL^−1^. For unfolding experiments, three different concentrations of SDS were chosen from transitions identified from ITC studies of SDS in bLG (2.0, 4.1, and 10.5 mM)^29^ together with an intermediate concentration (7.3 mM). Using concentrations of free SDS, as determined in,^29^ this gives a molar ratio (protein : SDS_bound_) of 1 : 12, 1 : 22, 1 : 43, and 1 : 63. For refolding experiments, solutions containing 4 mg mL^−1^ protein and a protein : SDS_bound_ molar ratio of 1 : 43 (17.0 mM SDS) were mixed with different concentrations of C_12_E_8_ so that the C_12_E_8_ end concentrations was just high enough for refolding (10.4 mM) or well above the critical concentration necessary for refolding (19.8 mM).^10^ All solutions were buffered in 10 mM NaHPO_4_ pH 7.0, 50 mM NaCl. The data were fitted with either single, double, or triple exponential decay functions: 
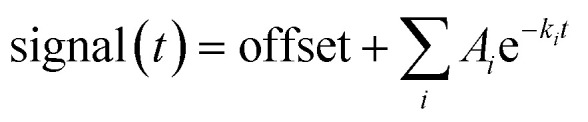
 where *A*_*i*_ is the amplitude of the *i*'th decay and *k*_*i*_ is the rate constant of the *i*'th decay.

### Small-angle X-ray scattering

All protein solutions were measured at a final concentration of 2 mg mL^−1^ and all solutions were in 10 mM NaHPO_4_ pH 7.0, 50 mM NaCl. Stopped-flow measurements were mixed in a 1 : 1 volume ratio. SDS and C_12_E_8_ concentrations were chosen as described above. Measurements were performed on the ID02 TRUSAXS beamline at the European Synchrotron (Grenoble, France) with an SFM-400 stopped-flow apparatus (Bio-Logic Science Instruments, France).^47^ Data was acquired in a quartz capillary with a diameter of 1.5 mm. A volume of 0.15 mL was injected from each syringe with a flow rate of 8 mL s^−1^ giving a pushing time of 40 ms and a mixing time of ∼3 ms. The deadtime was determined to 3.45 ms.

A buffer solution was used as background and water for conversion of the scattered intensity to absolute scale, *i.e.*, *I*(*q*) in cm^−1^. For all experiments the sample-to-detector distance was 3.0 m and all measurements were carried out at room temperature (approx. 21 °C). 2D SAXS scattering patterns were collected using a Rayonix MX-170HS CCD detector.^48^

Before measurements, the samples were investigated for radiation damage where a constant exposure of >250 ms was found to damage the protein samples. 30–50 time frames, each of duration 10–20 ms, were collected for each sample. An exponentially growing wait time between measurements was used to acquire most frames right after mixing while increasing the delays towards the end of the experiment and still avoiding radiation damage. No radiation damage was observed with the used acquisition times. The time stamp used for each frame is calculated as the average time during a single frame. Data is shown as a function of the scattering vector momentum, given by *q* = 4π*λ*^−1^ sin(*θ*) where the wavelength *λ* = 0.995 Å (photon energy: 12.46 keV) and 2*θ* is the scattering angle.

### Modelling

For the pure protein, the mass calculated from the forward scattering showed that the protein solution consisted of both monomers and dimers. The scattering of the crystal structure of bLG (PDB entry 3NPO^16^) and the dimeric crystal structure of bLG (PDB entry 1BEB^33^) were calculated on absolute scale using the Debye equation^49^ and in-house software that includes the hydration layer of the protein.^50^ Subsequently, a linear combination was fitted to the SAXS data providing the monomer and dimer fractions of the sample. Data from SDS and C_12_E_8_ micelles were modelled on absolute scale with a core–shell ellipsoid of revolution with the form factor from Guinier^51^ in a similar way as (^6,52^). The core contains the alkyl chains and the shell contains the head groups and also some water. Note that a sphero-cylinder model is also a plausible model, however, as scattering from small globular micelles are conventionally described by ellipsoidal models, this model has also been chosen in the present work.

For the complexes, an ellipsoidal model was also used. In this case, the shell contains the SDS head groups, the protein, and also some water. The application to the early stages of interaction with SDS required the modification of the ellipsoidal model, so that the center of the core and shell do not coincide. The form factor of this structure can be derived using the expressions in.^34^ For an ellipsoid of revolution with displaced center in the direction perpendicular to the symmetry axis (as indicated by the parameter *s*, see below), one has:
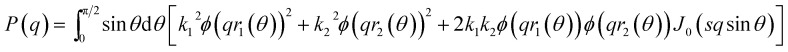
where:
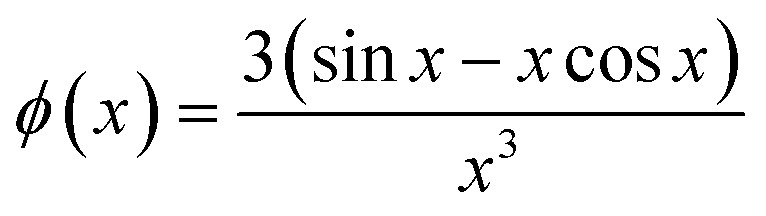
*J*_0_(*x*) is the Bessel function of first kind and zeroth order, *s* is the distance between the centers of core and shell, *r*_*i*_^2^(*θ*) = *R*_*i*_^2^(sin *θ*^2^ + *ε*_*i*_^2^ cos *θ*^2^), where *R*_*i*_ is the radius of the outer surface (*i* = 1) or core (*i* = 2) ellipsoid, and *ε*_*i*_ is the corresponding axis ratio. To keep the shell thickness uniform we set *ε*_2_ = (*ε*_1_*R*_1_ − *D*_shell_)/(*R*_1_ − *D*_shell_). The prefactors *k*_*i*_ are related to contrasts and volumes: *k*_1_ = *v*_1_Δ*ρ*_1_ where *v*_1_ = 4π*ε*_1_*R*_1_^3^/3 and Δ*ρ*_1_ is the excess scattering length density of the shell. For the core *k*_2_ = *v*_2_(Δ*ρ*_2_ − Δ*ρ*_1_) where *v*_2_ = 4π*ε*_2_*R*_2_^3^/3 where Δ*ρ*_2_ is the excess scattering length density of the core. Note that *s* should fulfil: *s* ≤ *R*_1_ − *R*_2_. The values used for the contrasts and volumes of the various components, based on literature values^6,53,54^ are given in the ESI.† Note that these values are based on independent measurements of partial specific densities and that the radii are fit parameters. The volume calculated from core radius divided by the volume of a C12 alkyl chain gives the aggregation number of the micelle. The total scattering length of the shell is calculated as the sum of that of the head groups and the protein and this is uniformly distributed in the shell, by comparing the volume of the shell with that of the head groups and the protein in shell, it is found that the shell also contains water (which has zero excess scattering length and thus contributes only to the volume and not to the total scattering length).

A random flight structure factor was used to describe the clustering of complexes:^34,35^
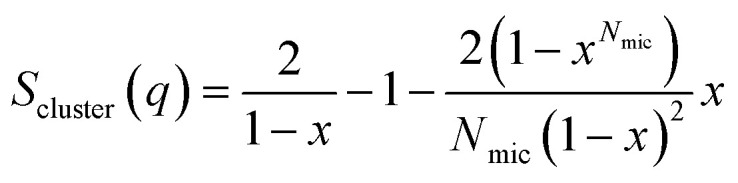
where *x* = sin(*qD*_mic_)/(*qD*_shell_), *D*_mic_ is the distance between micelle centers, and *N*_mic_ is the number of micelles in the cluster. For a non-integer value of *N*_mic_, a weighted sum of [*N*_mic_] and [*N*_mic_ + 1], where [*N*_mic_] is the largest integer smaller than *N*_mic_, was used. The structure factor was multiplied on the scattering form factor of the complex.

For some of the unfolding data sets, indirect Fourier transformations were also done^55,56^ in order to obtain the pair distance distribution functions, *p*(*r*), which is a histogram of distances between pairs of points, weighted by the excess scattering length density in these two points. The functions approach zero at the maximum diameter, *D*_max_, of the particles and gives some model-independent information on the shape of the particles. Additional information on radius of gyration, *R*_g_, and forward scattering, *I*(0), was obtained for the complete unfolding series by performing Guinier fits to the low-*q* region. *I*(0) is dominated by the scattering from the protein, and thus, *I*(0) is approximately proportional to the protein mass.

The refolding data were first attempted fitted using the core–shell model with a displaced center of the core, as described above, to which the SAXS data of mixed micelles were added with a variable scale factor. As this approach did not provide satisfactory fits, a linear combination of measured data of the pure species was fitted to the data. The linear combination contained: (1) The protein–SDS complex, (2) mixed micelles, (3) native protein, and a constant background. The mixed micelle scattering was only weakly dependent on the amount of SDS and therefore the scattering of micelles with the overall composition of surfactant was used throughout the series. The expression used for the fitting was:*I*_lin_(*q*) = *a*_1_*I*_1_(*q*) + *a*_2_*I*_2_(*q*) + *a*_3_*I*_3_(*q*) + *a*_4_where *a*_*i*_ are a fit parameters and *I*_*i*_(*q*) are the measured data sets for the various species. The parameters were optimized in a two-step procedure using a non-linear least-squares routine. In the first step the usual reduced chi-squared was used to estimate the goodness of fit:
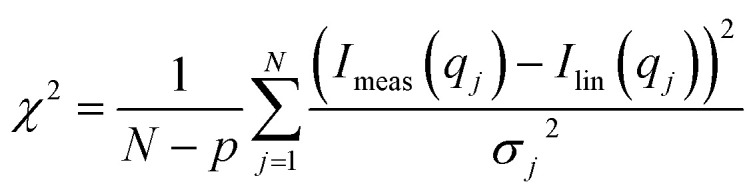
where *I*_meas_(*q*_*j*_) are the measured data points, which has the standard error *σ*_*j*_ from counting statistics, *N* is the number of data points, and *p* (=4) is the number of fit parameters. The basis functions *I*_*i*_(*q*) also have noise from counting statistics which was included in the next step by replacing *σ*_*j*_^2^ in the expression for chi-squared by:*σ*_*j*_^2^ = *a*_1_^2^*σ*_1,*j*_^2^ + *a*_2_^2^*σ*_2,*j*_^2^ + *a*_3_^2^*σ*_3,*j*_^2^where *σ*_*i*,*j*_^2^ is the standard error on the *j*'th point in the *i*'th basis function. The samples used for measuring the basis functions had known concentrations and with proper normalization conservation of protein mass would require that: *a*_1_ + *a*_2_ = 1; however, the scale factors added up to numbers lower than one, leading to the conclusion that a protein contribution was missing. Inspections of the fits suggested that the extra species of proteins had to be monomeric. It was assumed in the final model that it was a partly unfolded state and was correspondingly described by the scattering form factor of a Gaussian chain^57^ with a prefactor of 0.0244 cm^−1^ corresponding to the forward scattering of monomeric bLG at 2 mg mL^−1^. The expression used was:*I*_lin_(*q*) = *a*_1_*I*_1_(*q*) + *a*_2_*I*_2_(*q*) + *a*_3_*I*_3_(*q*) + *a*_4_*I*_4_(*q*) + *a*_5_where:
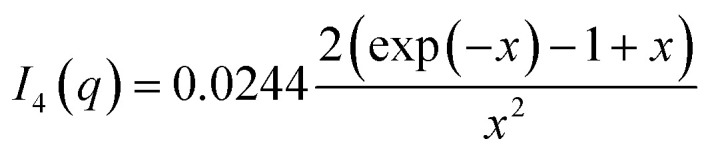
with *x* = *q*^2^*R*_g_^2^, where *R*_g_ is the root-mean-square ensemble-averaged radius of gyration. Conservation of protein total mass was imposed using: *a*_1_ = 1 − *a*_3_ − *a*_4_ in the fit expression. Reduced chi-squared values of the final fits were calculated as outlined above taking into account the errors on the experimental basis functions, additionally including the constraint of mass conservation.

## Associated content

Tryptophan fluorescence and circular dichroism of bLG in presence of SDS and C_12_E_8_; SF-SAXS data of bLG mixed with SDS; *p*(*r*) functions of SF-SAXS data; *R*_g_ and *I*(0) obtained from Guinier fits of SF-SAXS data; fitting of SAXS unfolding kinetics data using a linear combination of data from pure bLG, pure SDS, and bLG-SDS complex and corresponding *χ*^2^ values; *χ*^2^ of final fits to SAXS unfolding kinetics data; *χ*^2^ and protein scale factors from fits to SAXS refolding kinetics data when using a linear combination of data from mixed micelles, native bLG, and bLG-SDS complex; radius of gyration obtained from the Gaussian random chains included in fitting SAXS refolding kinetics data; *χ*^2^ of final fits to SAXS refolding kinetics data. Values of contrast and molecular volumes and other parameters used in the modelling of the unfolding data.

## Conflicts of interest

There are no conflicts to declare.

## Supplementary Material

SC-011-C9SC04831F-s001
