# Supplementary material for: A complete picture of protein unfolding and refolding in surfactants
Source: Chem Sci. 2019 Nov 22;11(3):699–712. doi: 10.1039/c9sc04831f (PMC8145811; doi:10.1039/c9sc04831f)
Supplement: SC-011-C9SC04831F-s001 [file SC-011-C9SC04831F-s001.pdf]

Electronic Supplementary Material (ESI) for Chemical Science.
This journal is © The Royal Society of Chemistry 2019

A complete picture of protein unfolding and refolding in surfactants

Jannik Nedergaard Pedersen[1], Jeppe Lyngsø[1], Thomas Zinn[2], Daniel Erik Otzen[3] and Jan Skov Pedersen[1]

[1]Interdisciplinary Nanoscience Center (iNANO) and Department of Chemistry, Aarhus University, Gustav Wieds Vej 14, 8000 Aarhus C, Denmark
[2]ESRF-The European Synchrotron, 38043 Grenoble, France
[3]Interdisciplinary Nanoscience Center (iNANO), Department of Molecular Biology and Genetics, Aarhus University, Gustav Wieds Vej 14, DK – 8000 Aarhus C, Denmark

Supporting Information

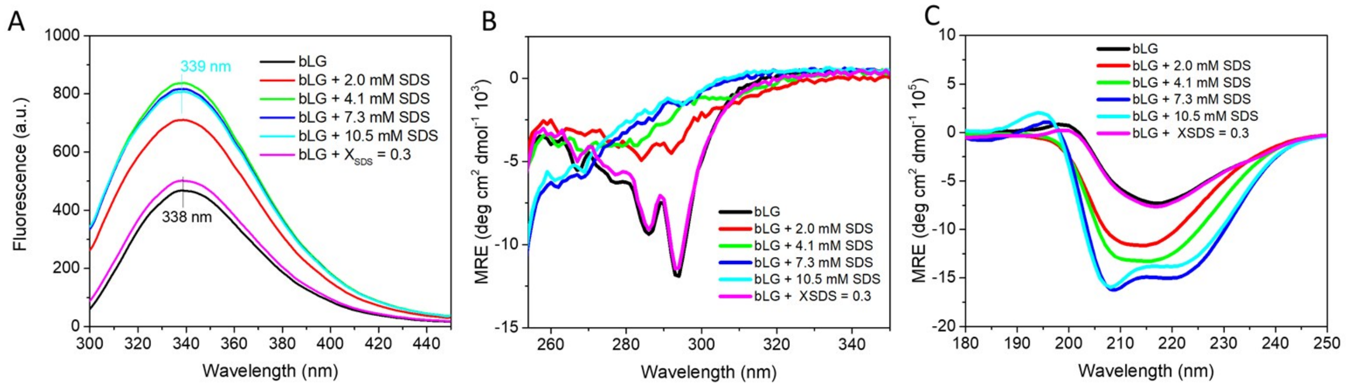

Fig. S1. (A) Tryptophan fluorescence measurements done with excitation at 280 nm of β-lactoglobulin (2 mg/ml) with SDS (8.5 mM), or SDS and $C_{12}E_8$ (19.8 mM). λ_{max} values are shown. (B) Equilibrium near-UV CD of bLG with SDS and $C_{12}E_8$. (C) Equilibrium far-UV CD of bLG with SDS and $C_{12}E_8$.

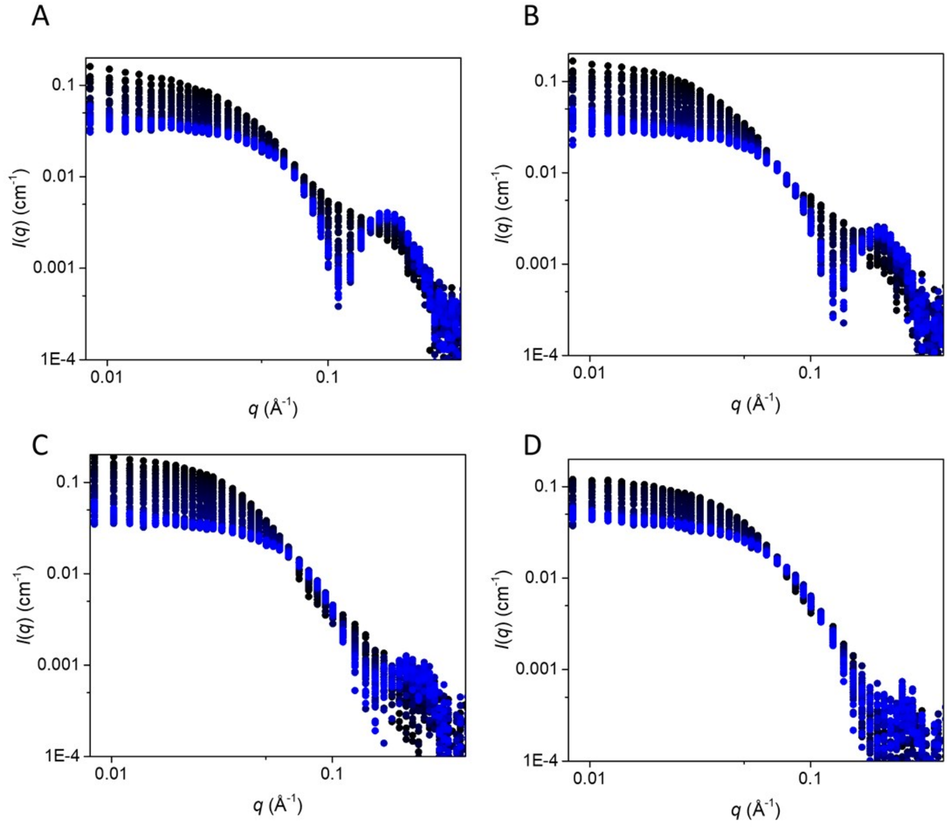

Fig. S2. Stopped-flow SAXS data sets of bLG mixed with (A) 10.5 mM SDS, (B) 7.3 mM SDS, (C) 4.1 mM SDS, and (D) 2.0 mM SDS with logarithm spaced times from ~4 ms to 12.5 min.

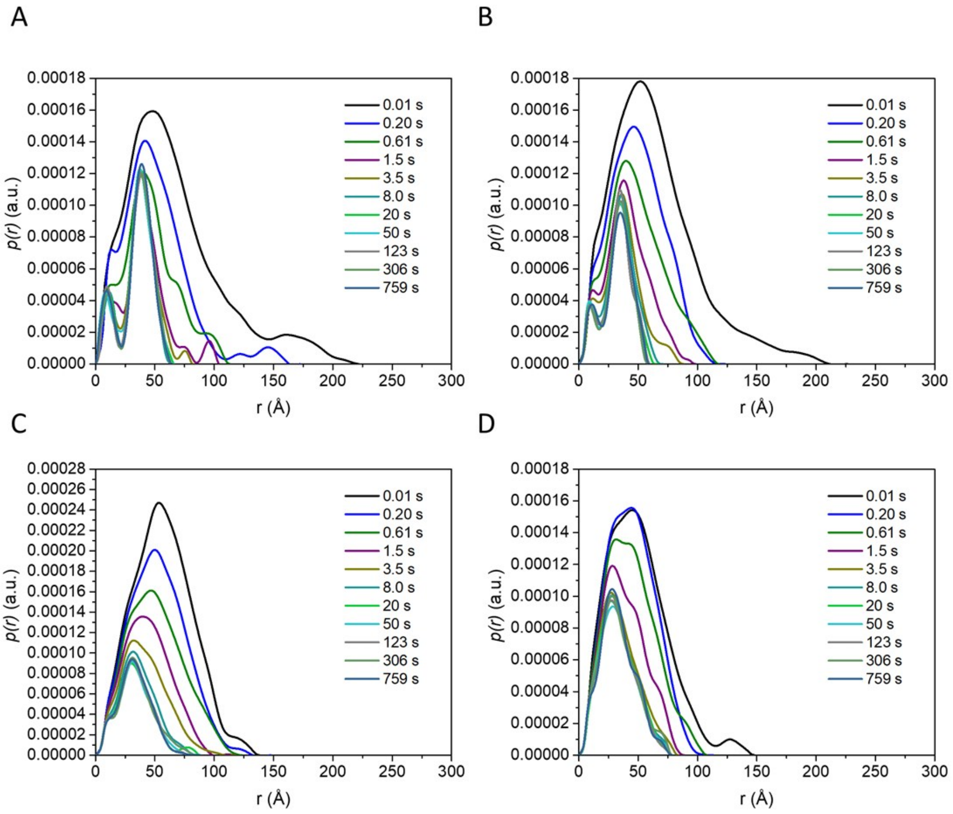

Fig. S3. $p(r)$ functions at selected times from the stopped-flow SAXS data of bLG mixed with (A) 10.5 mM SDS, (B) 7.3 mM SDS, (C) 4.1 mM SDS, and (D) 2.0 mM SDS. The maximum diameter decreases with time.

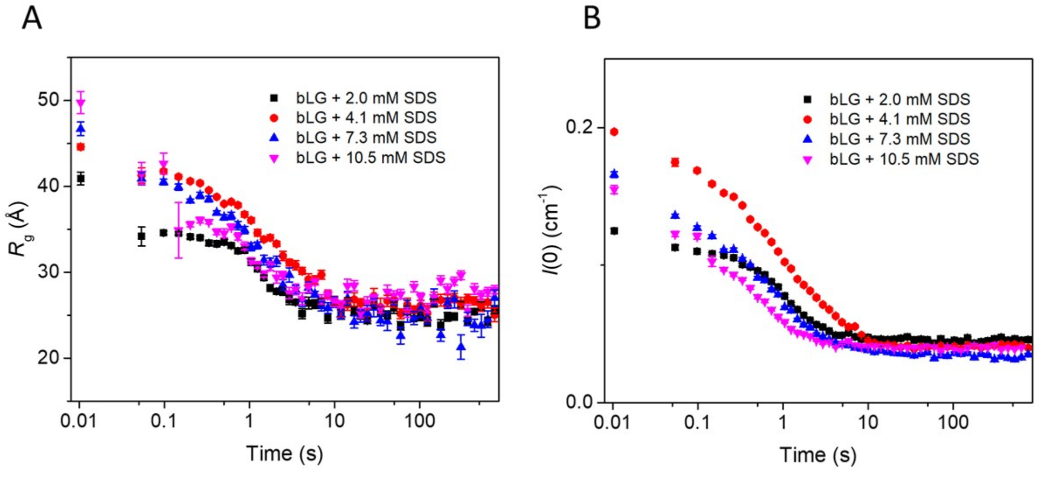

Fig. S4. (A) Radius of gyration R_g and (B) forward scattering $I(0)$ as determined from Guinier fits of the stopped-flow SAXS data of bLG mixed with different concentrations of SDS.

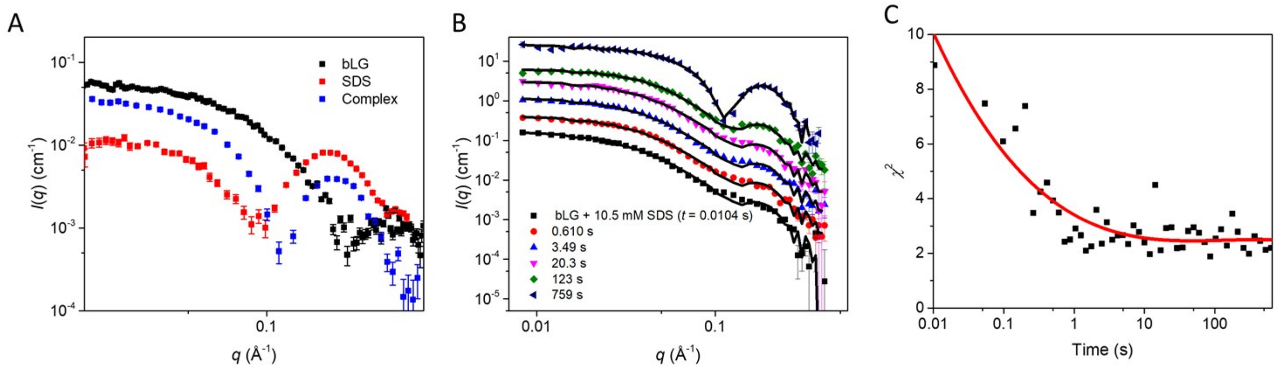

Fig. S5. (A) SAXS data of bLG (4 mg/ml), SDS (25 mM), and bLG-SDS complex (2 mg/ml bLG and 10.5 mM SDS) used for fitting unfolding data with a linear combination. (B) Selected frames from SAXS measurements of refolding kinetics of bLG (2 mg/ml) in SDS (10.5 mM). Lines represent fits using a linear combination of data seen in (A). For clarity, the data has been scaled with a factor of 3 for every time step. (C) χ^2 from fitting kinetic data in (B) to a linear combination of data in (A).

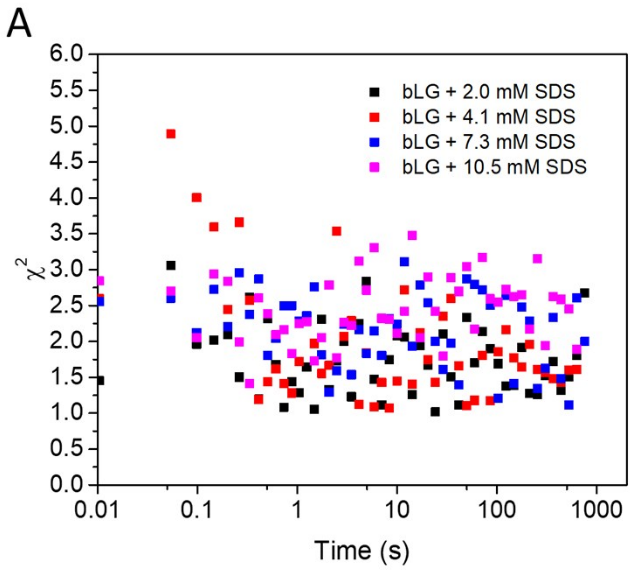

Fig. S6. (A) χ^2 values for fits to the core-shell model with an adjustable core offset for the stopped-flow unfolding SAXS data (Fig. 4).

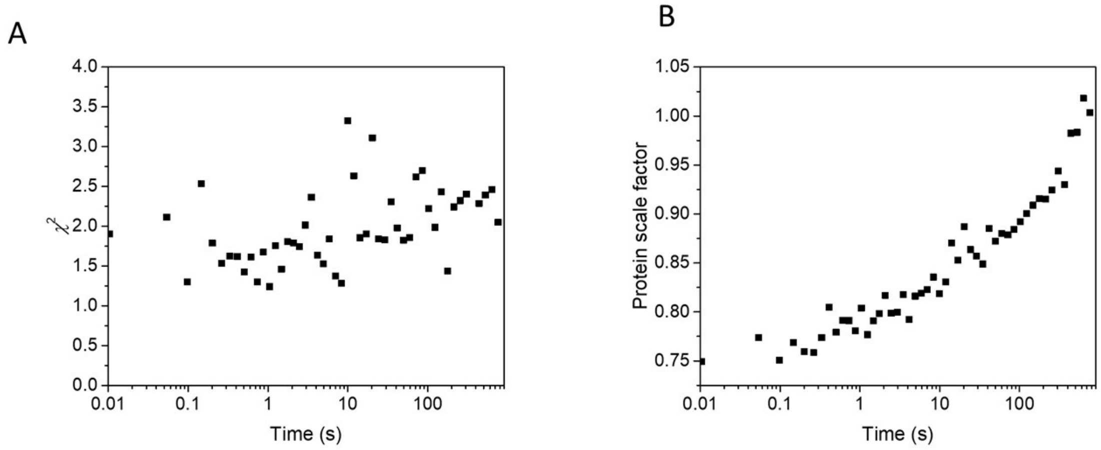

Fig. S7. (A) χ^2 from fitting SAXS data of refolding in χ_{SDS} = 0.45 to a linear combination of (1) the unfolded bLG-SDS complex, (2) folded bLG (77% dimer), and (3) mixed SDS-$C_{12}E_8$ micelles. (B) The sum of scale factors from the bLG-SDS complex and folded bLG as a function of time when a linear combination has been fitted to SAXS data of refolding in χ_{SDS} = 0.45.

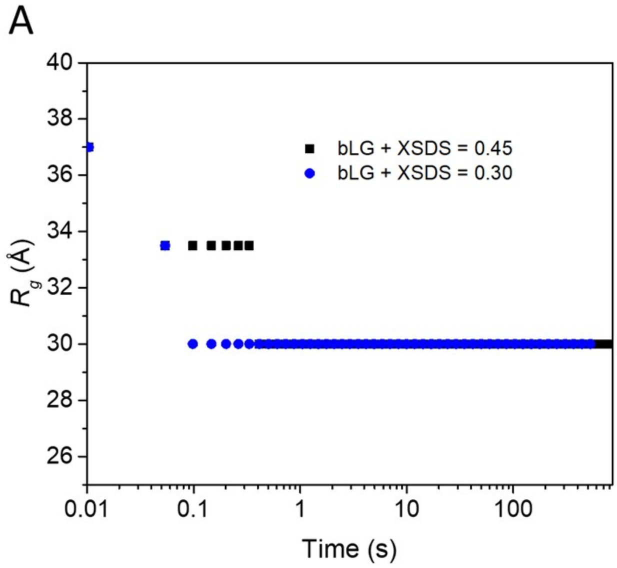

Fig. S8. Radius of gyration (R_g) of the Gaussian random chain used to fit data in Fig 7.

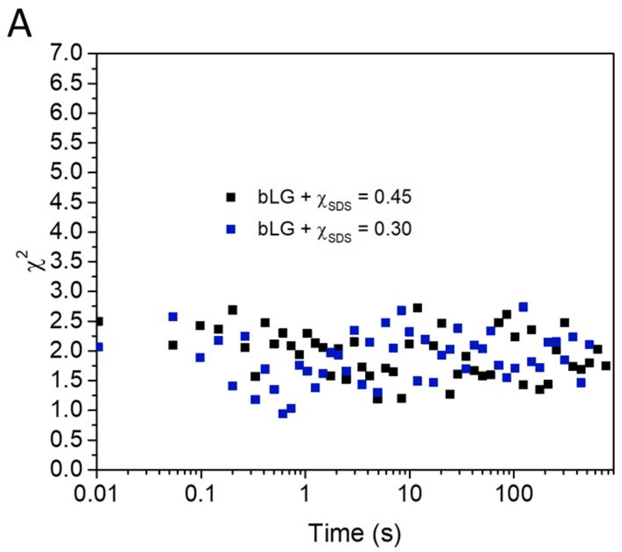

Fig. S9. (A) χ^2 values for fits to the data in Fig. 7A and 7B using a linear combination of native protein, random coil protein, bLG-SDS complex, and mixed SDS-$C_{12}E_8$ micelles, for fitting.

Table S1. Electron densities, ρ, excess electron densities, $\Delta\rho$, and partial volumes, ν, used in the model fitting. The scattering length densities are obtained by multiplying by the classical Thomson radius, 0.282 x 10^{-12} cm. For the protein, the scattering length per mass, $\Delta\rho_m$, and the partial specific volume, v_p, are given.

	ρ (e/Å^3)	$\Delta\rho$ (e/Å^3)	ν (Å^3)
Water	0.334		29.92
C12	0.275	-0.0592	353.0
SDS headgroup	0.975	0.551[i]	60.5
EO_8 headgroup	0.392	0.0579	61.2 x 7.5=459[ii]
	$\Delta\rho_m$ (cm/g)	v_p (cm^3/g)	
Protein	2.0 x 10^{10}	0.720	

[i] Scaled by 0.86 in order to fit the SAXS data for pure SDS micelles.

[ii] The number of EO units was used as a fit parameter and it was determined to be 7.5±0.3.
